# Revealing the effect of seed phosphorus concentration on seedling vigour and growth of rice using mutagenesis approach

**DOI:** 10.1038/s41598-022-04983-9

**Published:** 2022-01-24

**Authors:** Poli Yugandhar, Nallamothu Veronica, D. Subrahmanyam, P. Brajendra, S. Nagalakshmi, Akanksha Srivastava, S. R. Voleti, N. Sarla, R. M. Sundaram, Amitha Mithra Sevanthi, A. K. Singh, Satendra K. Mangrauthia

**Affiliations:** 1 ICAR-Indian Institute of Rice Research, Hyderabad, 500030 India; 2 ICAR-National Institute for Plant Biotechnology, New Delhi, India; 3 ICAR-Indian Agricultural Research Institute, New Delhi, India

**Keywords:** Biochemistry, Biotechnology, Physiology, Plant sciences

## Abstract

The harvested plant products, specifically, the grains of cereals are major drivers of soil phosphorus (P) depletion. However, the breeding or biotechnology efforts to develop low P seeds have not been attempted because of possible adverse effects on seedling vigour and crop establishment. Several studies have contradictory observations on influence of seed P on seedling vigour. Lack of appropriate genetic material has been the major bottleneck in reaching the consensus. In this study, we used 30 EMS induced mutants of rice cultivar Nagina22 to understand the role of seed P on seedling vigour and associated physiological processes. Seedling vigour, morpho-physiological characteristics, acid phosphatases, alpha-amylase, and expression of P transporter genes were analyzed in seedlings obtained from seeds of high and low grain P mutants. The study suggests that seed P has a significant role on seedling vigour, chlorophyll content and photosynthesis process of young seedlings, and P transport from roots. Notably, we identified few mutants such as *NH4791*, *NH4785*, *NH4714*, *NH4663*, *NH4614*, and *NH4618* which showed least influence of low seed P on seedling vigour and other metabolic processes. Therefore, these mutants can be used in breeding programs aiming for development of low P grains. Also, these and other identified mutants can be used to decipher the genetic and molecular mechanisms regulating the differential response of seed P on germination, seedling vigour and several other physiological processes influencing the crop growth and establishment.

## Introduction

Prevalence of low phosphorus (P) condition is a challenge faced by 30% of the world’s arable soils that reflect on the crop health^1^. While some parts of the world overuse P fertilizers causing eutrophication of lakes and coastal waters, the majority of the developing world has limited access to P fertilizers that lead to poor crop health^2^. In India, 53% of the soils are categorised as ‘low’ in available P, 30% of the soils as ‘moderate’ and only 12% of soils having ‘high’ level of available phosphorus^3^.

Phosphorus is considered as a vital nutrient for the growth and development of plants and animals. The sustainable growth of agriculture relies on efficient and economic application of fertilizers, specifically P fertilizers, because of finite natural resource in the form of high-grade rock phosphates. The global P cycle is mainly driven by loss of P from the field due to soil erosion and its fixation that becomes unavailable to plants^4^ and causes substantial pollution, and removal of soil P in the form of harvested plant products^5^. Among the harvested products, cereal grains share maximum plant phosphorus^6^. Further, it is pertinent to note that P in rice grains is stored mainly in the form of phytate, which is considered as an anti-nutrient, limiting the bioavailability of micronutrients like iron and zinc^7^. Therefore, reducing the P reserves in cereal grains without compromising the yield and the seedling vigour in subsequent generation can be one of the most viable strategies to make the agriculture sustainable with respect to P fertilizers^5,8^. In case of rice, 60–80% of total plant P is reserved in grains at maturity^9^.

Breeding for low-P seed trait is not encouraged due to the early studies suggesting that seed P reserves are critical for seedling vigour, crop establishment and yield as it positively regulates root development, and acquisition of growth-limiting resources such as nutrient and water^10–14^. However, these studies were challenged by different groups suggesting that external soil P supplementation neutralizes the negative effects of seed P concentration on seedling vigour^12,15,16^. Therefore, soil P status but not the seed P status may be the major factor influencing seedling vigour^8,9,17–19^. The major arguments for getting contradictory results were based on the methods and materials used for these studies^15,17^. The low P seeds obtained from P deficient soils^20–22^ or different environment^8^ or hydroponic methods^16^ may lead to artefacts in seed quality and impair other parameters, besides the P concentration of the seeds. These studies certainly raise scientific questions that need further investigation: (1) Does the seed P level have any role in seedling vigour when seeds are sown in soil with sufficient or deficient P? (2) Can the depletion of P from soil be reduced by lowering the seed P concentration? (3) Can the mutants that have less seed P, but no compromise on seedling vigour be developed to facilitate the breeding programs? In order to address these questions, in the present study, using the EMS (ethyl methanesulfonate) induced mutants of rice cultivar Nagina22^23,24^, we identified the mutants having differential ability to store grain P when grown on low P soil condition. Notably, these mutants did not show any difference in grain P concentration when grown in soil with normal P levels. Thirty such mutants were used to analyze the effects of seed P concentration on seedling vigour and other physiological processes. Few potential mutants showing least effect of seed P concentration on seedling vigour and associated traits were identified which can be used in breeding programs aiming to minimize the seed P content thereby reducing the soil P depletion.

## Material and methods

### The plant and seed material

We identified 30 Nagina22 (N22) EMS induced mutants showing differential grain P levels (when grown in low P soil) after screening of 500 stabilized mutants (at M5 generation) for five growing seasons (Kharif 2014, 2015, 2016 and Rabi 2015, 2016). The mutants were screened in low P (P deficient plot, Olsen P of 1.8 kg/ha) and normal P (applied with recommended dose of P, Olsen P of 24 kg/ha) field plot at ICAR-Indian Institute of Rice Research^25–28^. The mutants are named as NH which stands for N22-Hyderabad followed by the mutant number. Seeds of 30 mutants and wild type N22 were collected from both low P and normal P plots. Among the low P plot harvested seeds, 20 mutants showing grain P > 1.60 mg/g DW were termed as high grain P(HGP) while 10 mutants showing grain P < 1.20 mg/g DW were termed as low grain P (LGP) mutants. These seeds were sown in 30 cm diameter pots filled with 8 kg normal P soil (Olsen P 24 kg/ha). Fifty seeds collected from low P and normal P plots of each mutant were sown in two replications (n = 100). The following observations were recorded at 12 and 24 days of germination except for the fluorescence parameters (Fv/Fm and ETR), which were recorded on the 24th day. All plant experiments were carried out in accordance with the institute *guidelines* and *necessary permission* was obtained to collect the rice seeds.

### Seedling vigour, dry weight, and root/shoot/grain P content

Root length was measured from the base of the shoot/root junction till the tip of the longest root. Shoot length was measured from the base of the shoot/root junction till the tip of the longest leaf with a graduated scale and was expressed in cm. Seedling Vigour Index (SVI) was calculated by Seedling vigour index (SVI) = [Mean root length (Lr) + Mean shoot length (Ls)] × Percentage of seed germination (GP) Zhao et al.^29^. The same samples were shifted to a hot air oven maintained at 70 °C for 72 h to record the dry weight and utilized for estimation of P content in root and shoot samples using the method described by Saheki et al.^30^. After harvesting, grains were dried under natural condition for 6 days and same samples were utilized for estimation of P content in grains using the method described by Hanson^31^.

### Chlorophyll content and chlorophyll fluorescence parameters

One gram of leaf tissue was cut into pieces, placed in a tube containing 25 ml of 80% acetone, and stored in dark for 2 days. Absorbance of the chlorophyll solution was measured using a UV–Vis double beam spectrophotometer (SPECTRASCAN 2600, CHEMITO) for chlorophyll a, b, and carotenoids at 663.2, 646.8, and 470 nm, respectively. Chlorophyll fluorescence parameters (Fv/Fm and ETR) were measured using a portable fluorometer (PAM-210, WALZ, EFFELTRICH, Germany) after pre-adapting the leaf samples in dark for 30 min.

### Root and shoot acid phosphatase assay

Enzyme activity was measured as described in our previous report^28^. Enzyme extract was prepared by homogenizing 0.1 g of shoot or root tissue in 5 ml of ice cold 100 mM citrate buffer, pH 5.2. The homogenized samples were then centrifuged at 10,000 rpm for 15 min at 4 °C. The supernatant was collected and placed in a new tube. The supernatant (0.1 ml) was taken as an enzyme source. Reaction mixture (3 ml) consisted 0.5 ml of 10 mM p-Nitrophenol phosphate as substrate, 0.4 ml citrate buffer and 0.1 ml of enzyme extract^32^. The mixture was incubated at room temperature for 10 min. The reaction was terminated by adding 2 ml of 200 mM sodium carbonate. The absorbance of the solution was measured at 405 nm and the obtained values were converted into nano moles by plotting values against a pNP (p-Nitrophenol) standard curve generated with assay reagents. The acid phosphatase activity was expressed in nM of p-Nitrophenol released/min/g fresh weight.

### Externally secreted acid phosphatase assay

Roots were kept in a small glass beaker containing 10 mM pNP and incubated at 30 °C for 30 min. Sodium hydroxide (1 ml of 0.25 M) was added to stop the reaction. Absorbance was measured at 412 nm. The values were converted into micromoles by plotting values against the pNP standard curve generated with assay reagents^33^. The details of method followed for measurement of enzyme activity are described in our previous study^28^.

### alpha-amylase activity

Germinated rice seedlings at 4 days and 8 days were washed gently with deionized double distilled water and cleaned with tissue paper. Seedlings were ground in 10 ml of 0.5 M phosphate buffer (pH 6.0) and the homogenate was filtered with double layered muslin cloth and the clear supernatant was used as enzyme source. The alpha-amylase activity was carried out as described by Miller^34^ with minor modifications. Briefly, 1 ml of enzyme extract was added in the reaction mixture comprising sodium acetate buffer (pH 5.6) with 1% starch, 1 ml of DNSA (2,4-Dinitro phenol salicylic acid), heating at 90 °C for 5 min followed by cooling to room temperature. Absorbance was measured at 540 nm and alpha-amylase activity was calculated by plotting the standard curve with known concentrations of maltose.

### Gene expression analysis

The total RNA was isolated from the root and shoot tissues by using RNeasy Plant Mini Kit (QIAGEN). Later the RNA was quantified by using spectrophotometer (NANODROP ND-1000, THERMO FISHER, USA). Normalized RNA (1 µg) was used for cDNA synthesis by using RT2 First Strand Kit (QIAGEN). The cDNA was used as a template for qRT-PCR (APPLIED BIOSYSTEMS).

The qRT-PCR reaction mix preparation was followed as described by Manimaran et al.^35^ with minor modifications. The 20 µl reaction mixture was prepared by mixing the cDNA with 10 µl of 2× SYBR premix (QIAGEN) and 2 µM of gene specific primers. The relative expression levels were calculated as described by Schmittgen et al.^36^. All the qRT-PCR experiments were carried out with three biological replications. The OsActin was used as an internal control. The Ct values of tested genes were normalized with the Ct values of the reference gene (Actin) as described by Mangrauthia et al.^37^. To obtain ΔΔCT values, ΔCT of control (seedlings of normal P plot seeds) was subtracted from the ΔCT of test samples (seedlings of low P plot seeds). Fold change expression was calculated by 2^−ΔΔCt^. The details of tested genes and primers are given in our previous report^28^.

### Statistical analysis

The data from the experiments were analyzed by performing ANOVA using a statistical computer package (Statistix Ver. 8.1). The differences were estimated using the LSD test.

## Results

**Figure 1:**
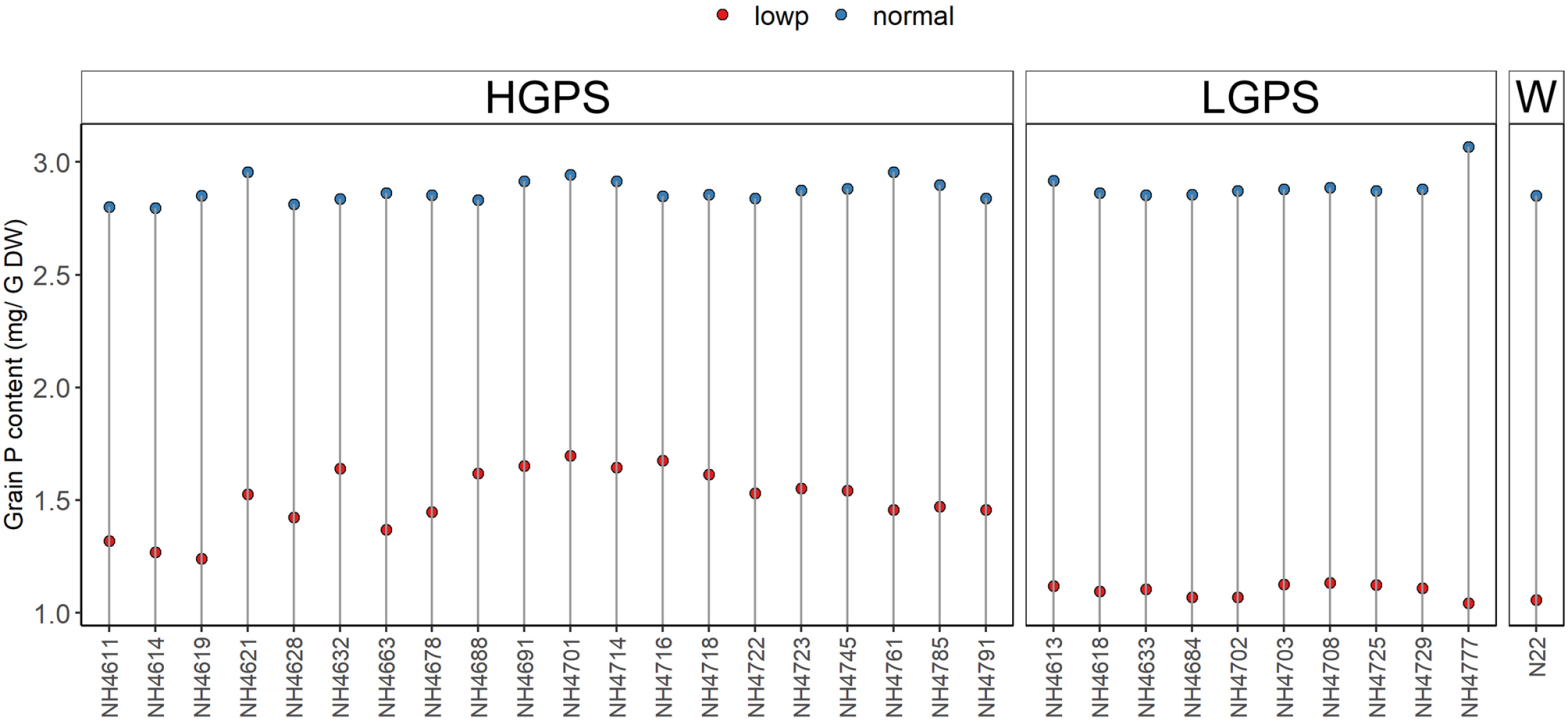
Initial grain P concentration in seeds collected from low P (shown in red dots) and normal P (shown in blue dots) filed plots.

Seeds from 20 HGP and 10 LGP mutants along with the wild type N22 were harvested from low soil P and normal soil P field plots. Before sowing these seeds, the initial grain P content was estimated in seeds obtained from low P (LP) and normal P (NP) conditions. The difference in grain P content was insignificant between NP-HGP and NP-LGP mutants. Notably, the grain P content was 27% more in LP-HGP mutants than LP-LGP mutants (Fig. 1). The seeds obtained from LP and NP conditions were germinated in normal P soil, and the seedlings were designated as LP-HGPS (low P plot seeds-HGP seedlings), LP-LGPS (low P plot seeds-LGP seedlings), NP-HGPS (normal P plot seeds-HGP seedlings), and NP-LGPS (normal P plot seeds-LGP seedlings). Various morphological and physiological parameters of these seedlings were analyzed to assess the differential response of seed P content in germinability and seedling growth.

### LP-HGPS show better seedling growth than LP-LGPS

**Figure 2:**
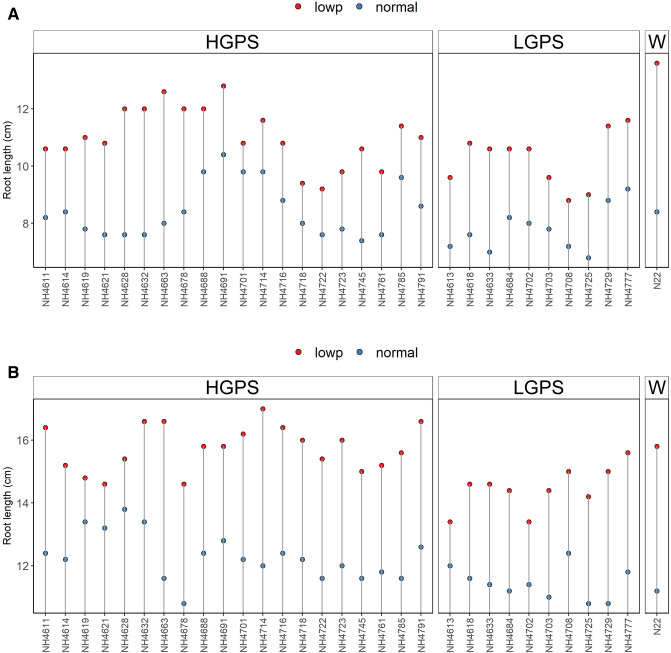
Root length of seedlings obtained from germination of seeds harvested from low P (shown as red dots) and normal P (shown as blue dots) fields. Wild type N22 (W), high grain P mutant seedlings (HGPS), and low grain P mutant seedlings (LGPS) were grown under normal P condition. (**A**) Root length at 12 days of germination. (**B**) Root length at 24 days of germination. The values represent the mean of 100 plants.

**Figure 3:**
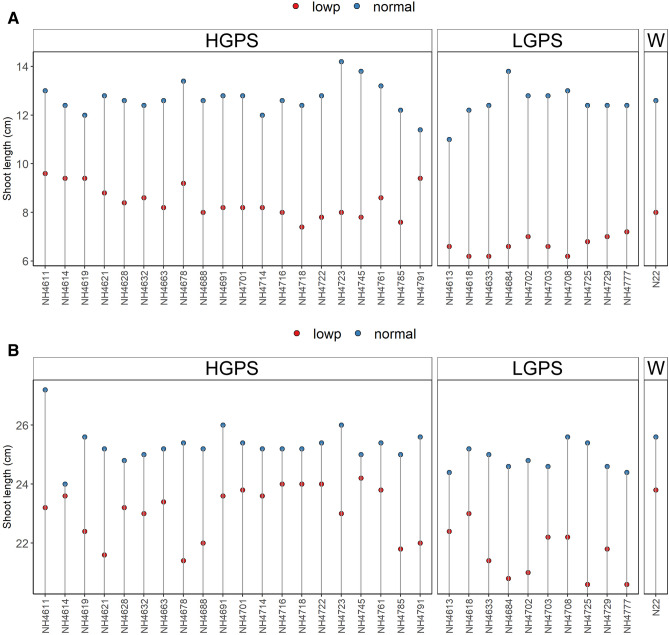
Shoot length of seedlings obtained from germination of seeds harvested from low P (shown as red dots) and normal P (shown as blue dots) fields. Wild type N22 (W), high grain P mutant seedlings (HGPS), and low grain P mutant seedlings (LGPS) were grown under normal P condition. (**A**) Shoot length at 12 days of germination. (**B**) Shoot length at 24 days of germination. The values represent the mean of 100 plants.

The root and shoot length of LP-HGPS was significantly more than LP-LGPS and N22. The mean root length of LP-HGPS and LP-LGPS was 11.04 cm and 10.26 cm at 12 days and 15.76 cm and 14.58 cm at 24 days, respectively. The mean root dry weight of LP-HGPS and LP-LGPS was 0.017 and 0.009 g at 12 days, and 0.024 and 0.022 g at 24 days, respectively. Notably, LP-HGPS, LP-LGPS and LP-N22 showed significant increase in root length as compared to NP-HGPS, NP-LGPS, and NP-N22 (Fig. 2). Shoot length was also recorded at 12 and 24 days of germination. NP-HGPS, NP-LGPS, and NP-N22 did not show any significant variation in shoot length, however, LP-HGPS showed higher shoot length than LP-LGPS at both the time points. The mean shoot length of LP-HGPS and LP-LGPS was 8.4 cm and 6.7 cm at 12 days, and 23 cm and 21.6 cm at 24 days, respectively (Fig. 3). Interestingly, one of the HGP mutants (*NH4614*) showed similar shoot length under LP and NP at 24 days of germination. In contrast, LGP mutants such as *NH4777*, *NH4684*, and *NH4725* showed severe impact on shoot length under LP when compared with NP.

**Figure 4:**
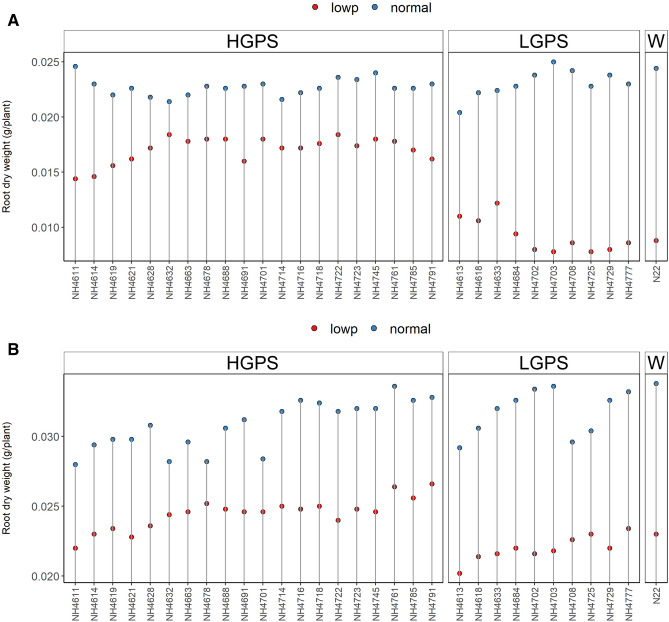
Root dry weight of seedlings obtained from germination of seeds harvested from low P (shown as red dots) and normal P (shown as blue dots) fields. Wild type N22 (W), high grain P mutant seedlings (HGPS), and low grain P mutant seedlings (LGPS) were grown under normal P condition. (**A**) Root dry weight at 12 days of germination. (**B**) Root dry weight at 24 days of germination. The values represent the mean of 20 plants.

**Figure 5:**
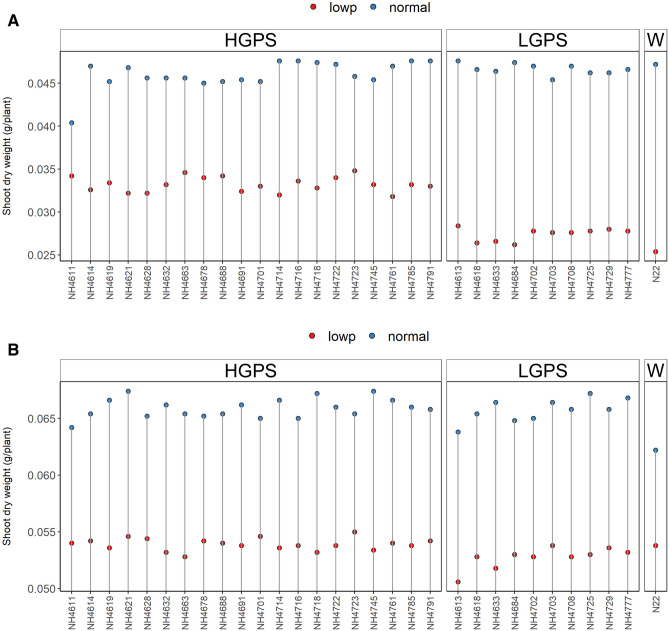
Shoot dry weight of seedlings obtained from germination of seeds harvested from low P (shown as red dots) and normal P (shown as blue dots) fields. Wild type N22 (W), high grain P mutant seedlings (HGPS), and low grain P mutant seedlings (LGPS) were grown under normal P condition. (**A**) Shoot dry weight at 12 days of germination. (**B**) Shoot dry weight at 24 days of germination. The values represent the mean of 20 plants.

NP-HGPS, NP-LGPS, and NP-N22 did not show significant variation in root and shoot dry weight, however, LP-HGPS showed higher root and shoot dry weight than LP-LGPS at both the time points (Figs. 4, 5). The average root dry weight of LP-HGPS and LP-LGPS was 0.017 and 0.009 g at 12 days, and 0.024 and 0.022 g at 24 days, respectively. LP-HGPS exhibited 89% and 10% more root dry weight than LP-LGPS at 12 and 24 days (Fig. 4). The average shoot dry weight of LP-HGPS and LP-LGPS was 0.033 and 0.027 g at 12 days, and 0.054 and 0.053 g at 24 days, respectively. LP-HGPS exhibited 22% and 2% more shoot dry weight than LP-LGPS at 12 and 24d, respectively (Fig. 5). The difference between LP-HGPS and LP-LGPS was more pronounced at 12 days than 24 days old seedlings.

### Seedling vigour

**Figure 6:**
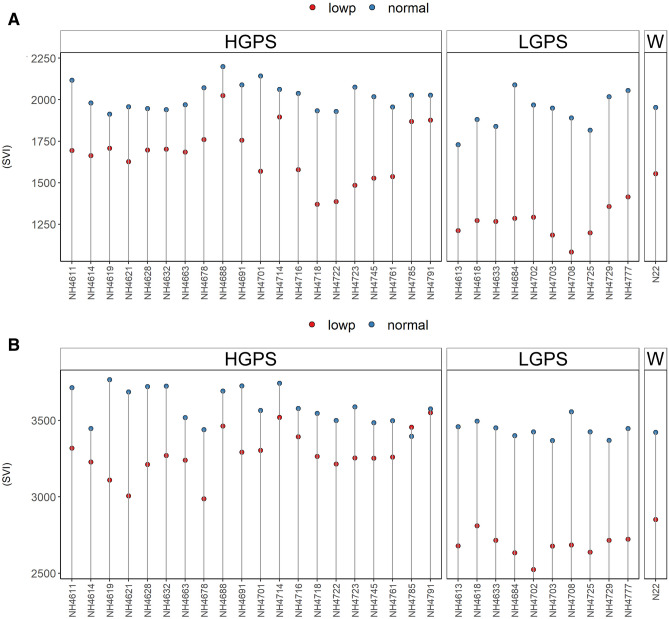
Seedling vigour index (SVI) of seedlings obtained from germination of seeds harvested from low P (shown as red dots) and normal P (shown as blue dots) fields. Wild type N22 (W), high grain P mutant seedlings (HGPS), and low grain P mutant seedlings (LGPS) were grown under normal P condition. (**A**) SVI at 12 days of germination. (**B**) SVI at 24 days of germination. The values represent the mean of 100 plants.

SVI was comparable in NP-HGPS, NP-LGPS, and NP-N22 at both the time points, but it was more severely affected in LP-LGPS than LP-HGPS (Fig. 6). The average mean SVI of LP-HGPS and LP-LGPS was 1651.8 and 1256.8 at 12 days and 3290.7 and 2680.1 at 24 days, respectively. Germination percentage of LP-HGPS and LP-LGPS was 85 and 74 percent, respectively. Notably, mutants *NH4785* and *NH4791* showed similar SVI under NP and LP conditions at 24 days of germination. Both of these mutants showed lesser effect of LP on SVI at 12 days of germination also. Among all the mutants, *NH4688* showed highest SVI under LP and NP at 12 days of germination.

### Chlorophyll (Chl) and fluorescence parameters

**Figure 7:**
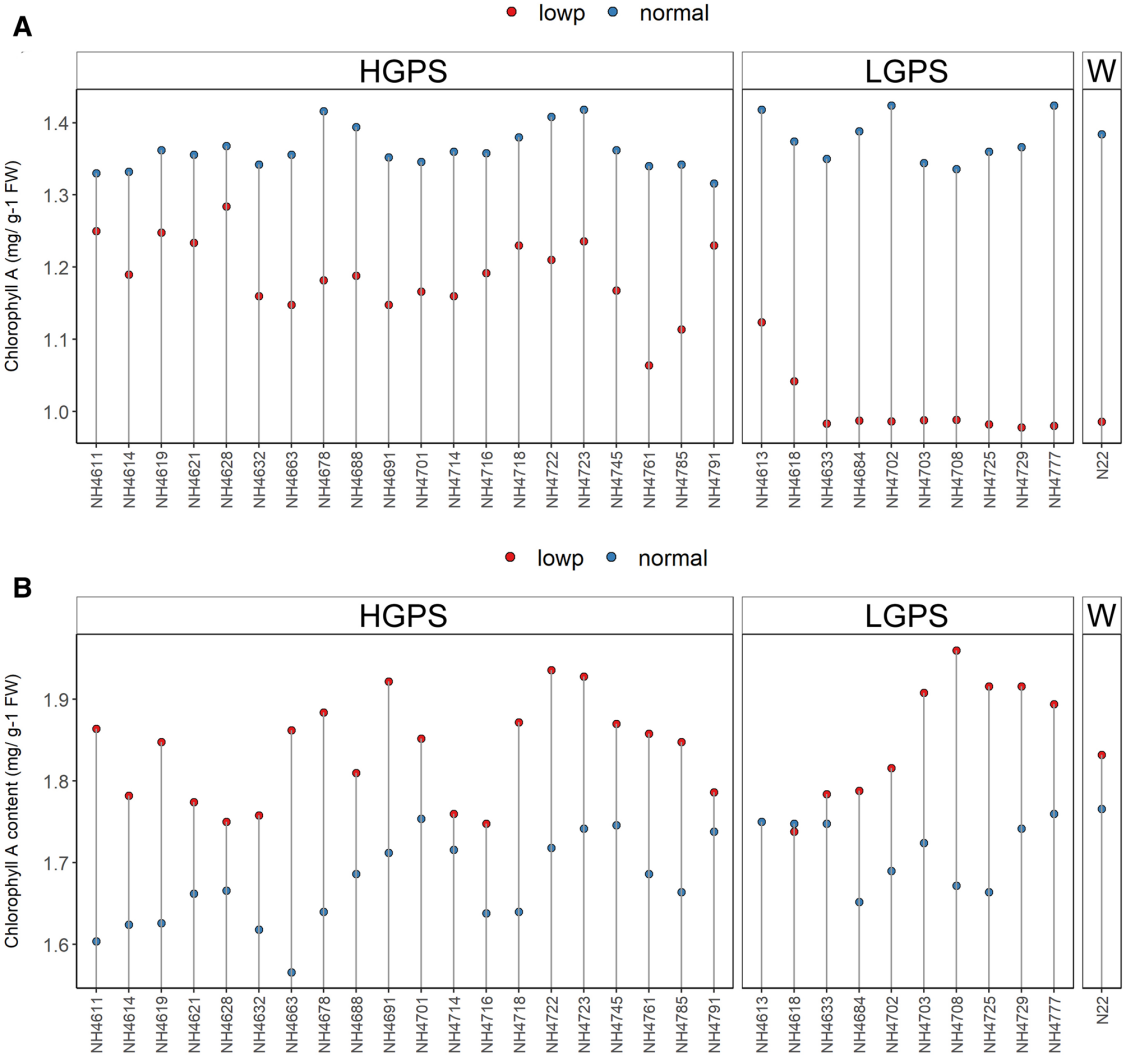
Chlorophyll a (Chl a) content of seedlings obtained from germination of seeds harvested from low P (shown as red dots) and normal P (shown as blue dots) fields. Wild type N22 (W), high grain P mutant seedlings (HGPS), and low grain P mutant seedlings (LGPS) were grown under normal P condition. (**A**) Chl a at 12 days of germination. (**B**) Chl a at 24 days of germination. The values represent the mean of 6 plants.

**Figure 8:**
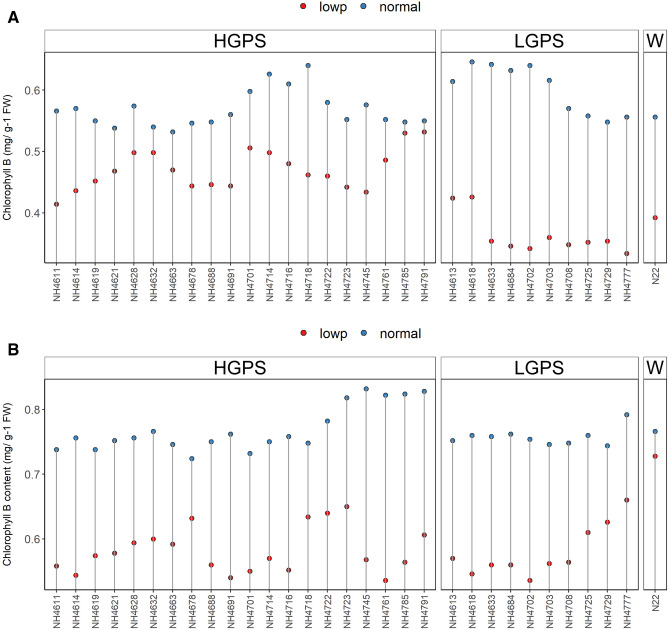
Chlorophyll b (Chl b) content of seedlings obtained from germination of seeds harvested from low P (shown as red dots) and normal P (shown as blue dots) fields. Wild type N22 (W), high grain P mutant seedlings (HGPS), and low grain P mutant seedlings (LGPS) were grown under normal P condition. (**A**) Chl b at 12 days of germination. (**B**) Chl b at 24 days of germination. The values represent the mean of 6 plants.

**Figure 9:**
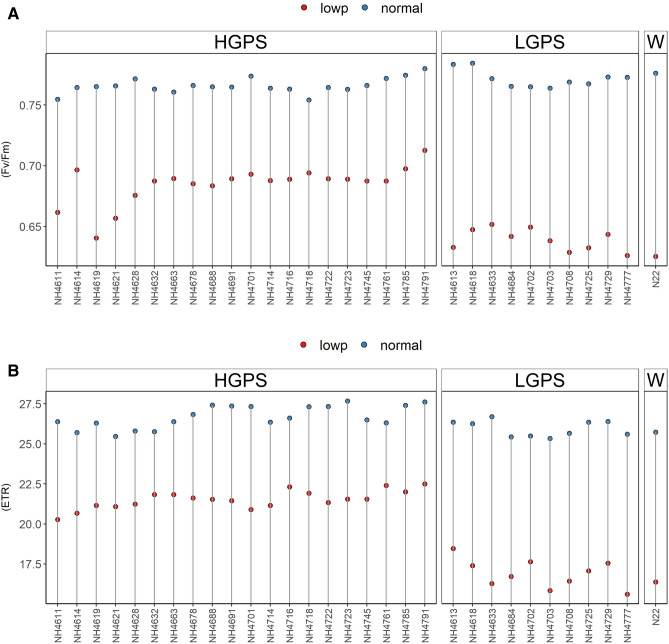
Fv/Fm and ETR of seedlings obtained from germination of seeds harvested from low P (shown as red dots) and normal P (shown as blue dots) fields. Wild type N22 (W), high grain P mutant seedlings (HGPS), and low grain P mutant seedlings (LGPS) were grown under normal P condition. (**A**) Fv/Fm at 24 days of germination. (**B**) ETR at 24 days of germination. The leaf samples at 24th day were collected and kept in dark for 30 min before measuring the Fv/Fm and ETR. The values represent the mean of 6 plants.

Reciprocal trend of Chl a content was observed at 12 and 24 days. At 12 days, all the mutants showed reduced Chl a under LP in comparison to NP, though the degree of reduction was more severe in LP-LGPS than LP-HGPS. On the other hand, at 24 days, all the mutants showed increased Chl a under LP in comparison to NP, and there was not much difference between LP-LGPS and LP-HGPS. Chl a did not show significant difference in NP-HGPS, NP-LGPS, and NP-N22 at both the time points (Fig. 7). LP-*NH4663* showed maximum gain of Chl a when compared with NP-*NH4663*, while NH4618 did not show difference under LP and NP conditions at 24 days. Chl b was reduced in all the mutants under LP condition at both the time points, however, the degree of reduction was more severe in LP-LGPS than LP-HGPS (Fig. 8). Mutants *NH4785* and *NH4791* showed similar Chl b content at 12 days under LP and NP conditions. Similarly, fluorescence parameters such as Fv/Fm and ETR were reduced in all the mutants under LP condition at 24 days. LP-LGPS showed more reduction of Fv/Fm and ETR than LP-HGPS (Fig. 9). Fv/Fm ranged from 0.64 to 0.71 in LP-HGPS and 0.63 to 0.65 in LP-LGPS, while ETR ranged from 20.28 to 22.50 in LP-HGPS and 15.62 to 18.46 in LP-LGPS. Fv/Fm and ETR were comparable in NP-HGPS, NP-LGPS, and NP-N22.

### P content in root and shoot tissues

**Figure 10:**
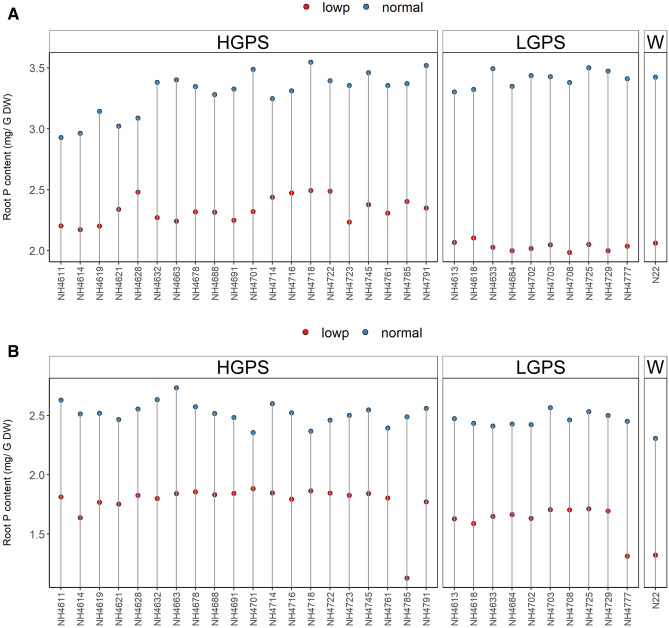
Root P content of seedlings obtained from germination of seeds harvested from low P (shown as red dots) and normal P (shown as blue dots) fields. Wild type N22 (W), high grain P mutant seedlings (HGPS), and low grain P mutant seedlings (LGPS) were grown under normal P condition. (**A**) Root P content at 12 days of germination. (**B**) Root P content at 24 days of germination. The values represent the mean of 6 plants.

**Figure 11:**
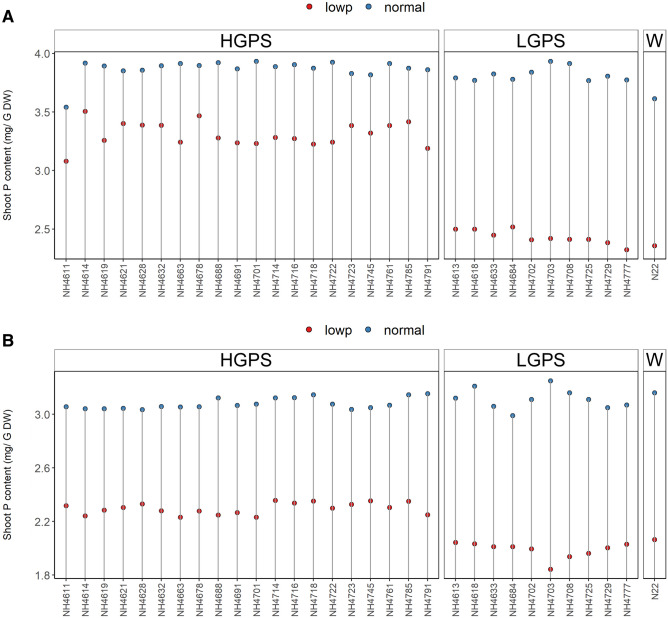
Shoot P content of seedlings obtained from germination of seeds harvested from low P (shown as red dots) and normal P (shown as blue dots) fields. Wild type N22 (W), high grain P mutant seedlings (HGPS), and low grain P mutant seedlings (LGPS) were grown under normal P condition. (**A**) Shoot P content at 12 days of germination. (**B**) Shoot P content at 24 days of germination. The values represent the mean of 6 plants.

The differences in root and shoot P content were significant between LP and NP seedlings and also between HGPS and LGPS at 12 and 24 days (Figs. 10, 11). Compared to NP, the P content was significantly less in LP seedlings in all the mutants but the degree of reduction was more in LP-LGPS than LP-HGPS. The root and shoot P content was 13% and 27% more in LP-HGPS than LP-LGPS at 12d. Similarly, at 24 days, LP-HGPS exhibited 10% and 14% more root and shoot P content than LP-LGPS. At 12 days, LP-HGPS showed 29% and 14% lesser P content than NP-HGPS in root and shoot while LP-LGPS showed 40% and 36% lesser root and shoot P content than NP-LGPS. At 24 days, LP-HGPS showed 29% and 2% lesser root and shoot P content than NP-HGPS while LP-LGPS showed 34% and 14% lesser root and shoot P content than NP-LGPS.

### Acid phosphatase activity

**Figure 12:**
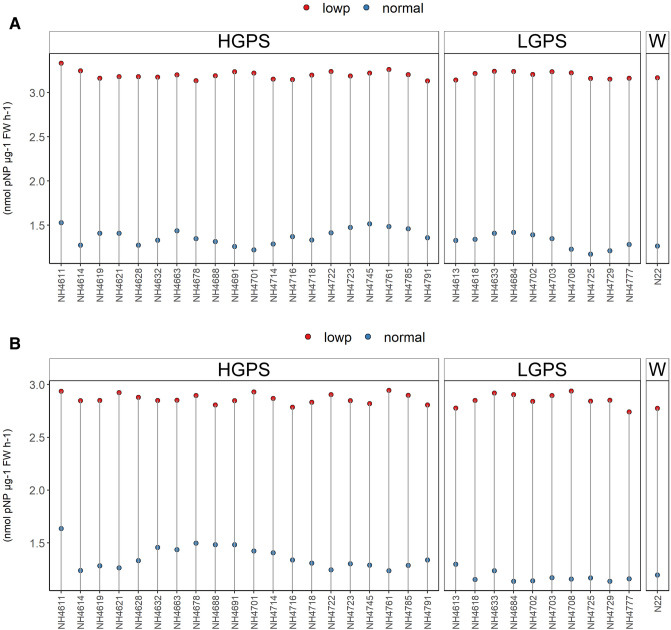
Externally secreted acid phosphatase (ESApase) enzyme activity of seedlings obtained from germination of seeds harvested from low P (shown as red dots) and normal P (shown as blue dots) fields. Wild type N22 (W), high grain P mutant seedlings (HGPS), and low grain P mutant seedlings (LGPS) were grown under normal P condition. (**A**) ESApase enzyme activity at 12 days of germination. (**B**) ESApase enzyme activity at 24 days of germination. The values represent the mean of 6 plants.

**Figure 13:**
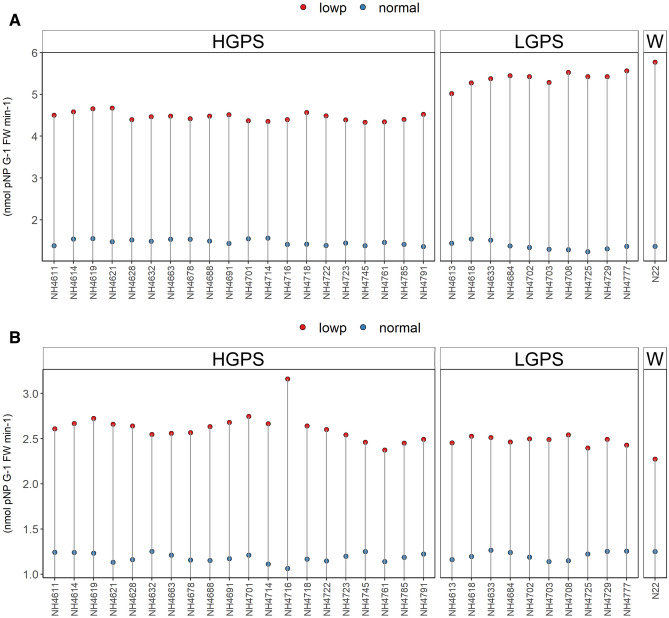
Root acid phosphatase (RApase) enzyme activity of seedlings obtained from germination of seeds harvested from low P (shown as red dots) and normal P (shown as blue dots) fields. Wild type N22 (W), high grain P mutant seedlings (HGPS), and low grain P mutant seedlings (LGPS) were grown under normal P condition. (**A**) RApase enzyme activity at 12 days of germination. (**B**) RApase enzyme activity at 24 days of germination. The values represent the mean of 6 plants.

**Figure 14:**
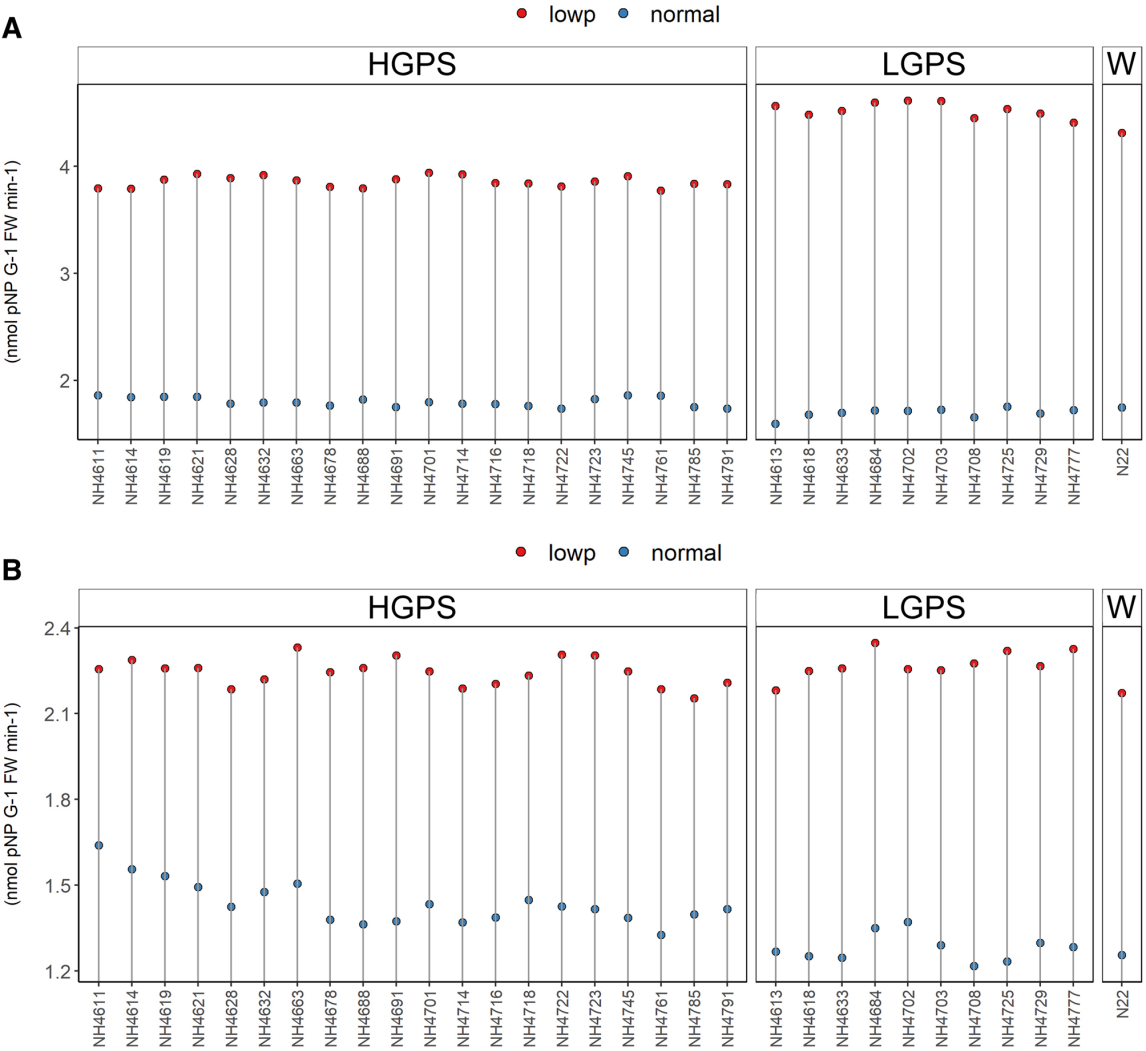
Shoot acid phosphatase (SApase) enzyme activity of seedlings obtained from germination of seeds harvested from low P (shown as red dots) and normal P (shown as blue dots) fields. Wild type N22 (W), high grain P mutant seedlings (HGPS), and low grain P mutant seedlings (LGPS) were grown under normal P condition. (**A**) SApase enzyme activity at 12 days of germination. (**B**) SApase enzyme activity at 24 days of germination. The values represent the mean of 6 plants.

Low P stress responsive enzymes such as externally secreted acid phosphatase (ESApase), root acid phosphatase (RApase), and shoot acid phosphatase (SApase) play a key role in mobilizing the organic P to available P in plants. The low P condition triggers these P responsive enzymes to compensate the P deficiency/P availability upto some extent. The ES Apase, RApase and SApase enzymes activity was induced in all the LP seedlings (Figs. 12, 13, 14). Though the percent increase (from NP to LP) of ESApase, RApase, and SApase enzyme activity was more in LGPS than HGPS, the differences in the ESApase enzyme activity among LP-HGPS, LP-LGPS and LP-N22 were not significant at both 12 days and 24 days, respectively (Fig. 12). The mean RApase enzyme activity in LP-HGPS, LP-LGPS and LP-N22 at 12d was 4.47, 5.38 and 5.77, respectively (Fig. 13). The mean RApase enzyme activity in LP-HGPS, LP-LGPS, and LP-N22 at 24 days was 2.62, 2.48 and 2.27, respectively. The mean SApase enzyme activity in LP-HGPS, LP-LGPS and LP-N22 at 12d was 3.86, 4.53 and 4.31, respectively (Fig. 14). The mean SApase enzyme activity in LP-HGPS, LP-LGPS, and LP-N22 at 24 days was 2.24, 2.27 and 2.17, respectively. Compared to 12 days old LP seedlings, the ESApase, RApase, and SApase activity was 10%, 47%, and 45% lower in 24 days old LP seedlings.

### Alpha-amylase activity

**Figure 15:**
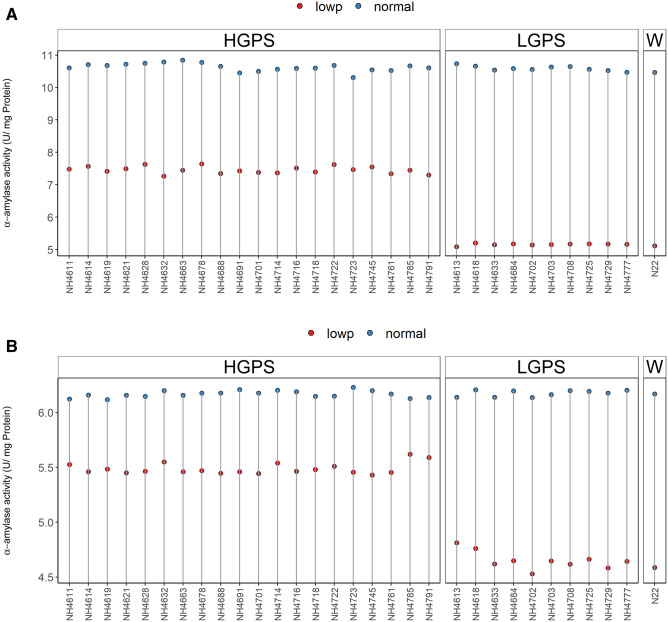
Alpha amylase enzyme (AAE) activity of seedlings obtained from germination of seeds harvested from low P (shown as red dots) and normal P (shown as blue dots) fields. Wild type N22 (W), high grain P mutant seedlings (HGPS), and low grain P mutant seedlings (LGPS) were grown under normal P condition. (**A**) AAE activity at 4 days of germination. (**B**) AAE activity at 8 days of germination. The values represent the mean of 10 plants.

Alpha-amylase enzyme (AAE) plays a significant role during the germination and later throughout the growth period of rice. AAE activity was comparable in NP-HGPS, NP-LGPS, and NP-N22 at both the time points (4 days and 8 days), but it was significantly high in LP-HGPS than LP-LGPS, and LP-N22 (Fig. 15). The average mean AAE of LP-HGPS and LP-LGPS was 7.46 and 5.16 at 4 days and 5.49 and 4.65 at 8 days, respectively. The AAE activity was significantly more in NP than LP condition in all the mutants and N22.

### Correlation studies

**Figure 16:**
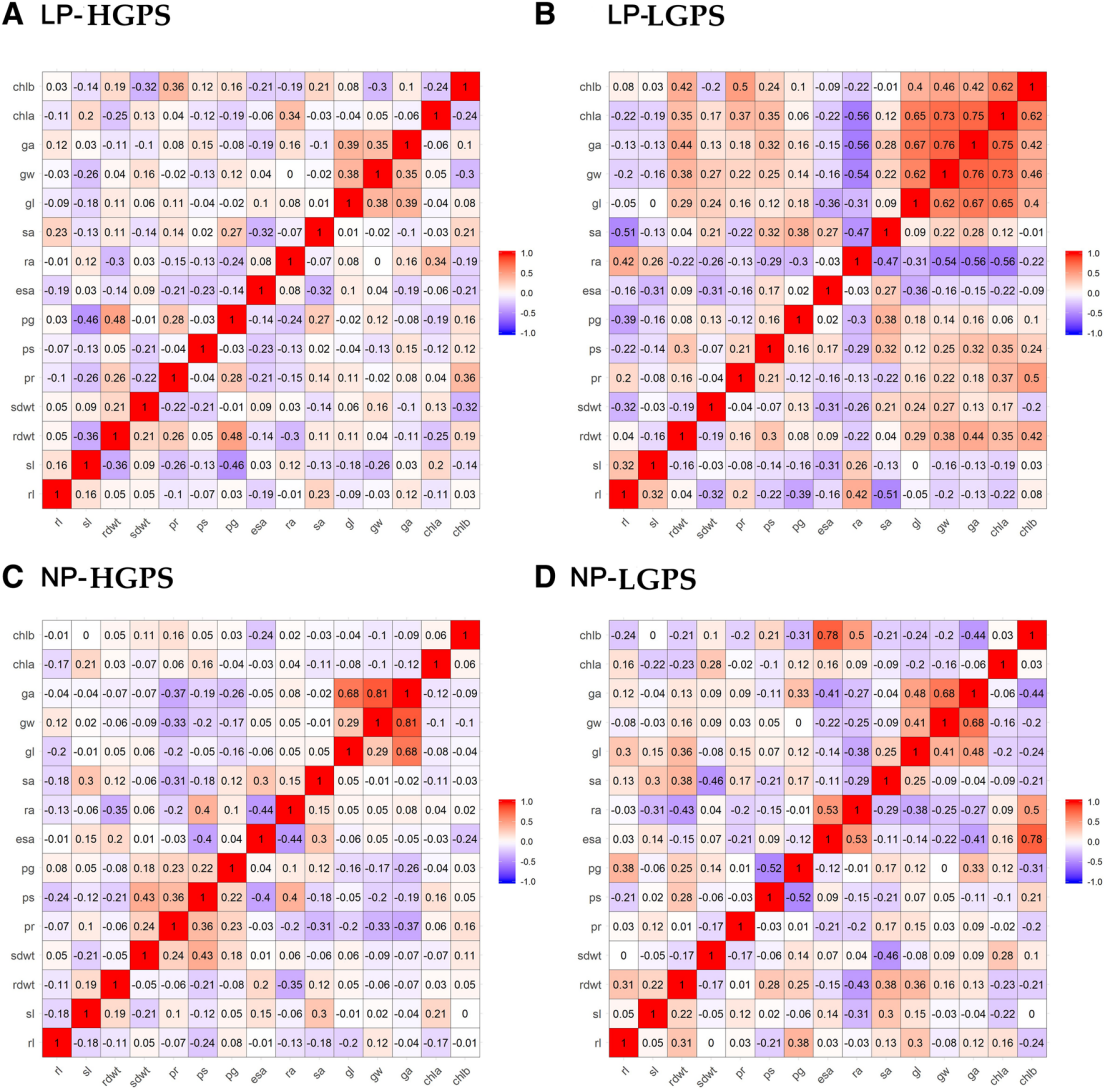
Correlogram of traits under LP and NP conditions in HGPS and LGPS at 12 days. The scale represents Pearson correlation values. Blue and red colours represent negative and positive correlation, respectively. root length (rl), shoot length (sl), root dry weight (rdwt), shoot dry weight (sdwt), P content in root (pr), P content in shoot (ps), P content in grain (pg), externally secreted acid phosphatase (esa), root acid phosphatase (ra), shoot acid phosphatase (sa), grain length (gl), grain width (gw), grain area (ga), chlorophyll A content (chla), chlorophyll B content (chlb).

Correlation analysis of various parameters taken from 12 days old seedlings was performed. In LP-HGPS, seedling vigour was positively and significantly correlated with germination percentage, root length, and shoot length (Fig. 16A). Root dry weight was positively correlated while shoot length was negatively correlated with grain P content. In LP-LGPS, root length was positively correlated with RApase but negatively correlated with SApase. Similarly, P content in root and root dry weight showed significant positive correlation with Chl b content. RApase showed significant negative correlation with Chl a content (Fig. 16B). These correlations were not noticed in NP-HGPS and NP-LGPS (Fig. 16C,D).

**Figure 17:**
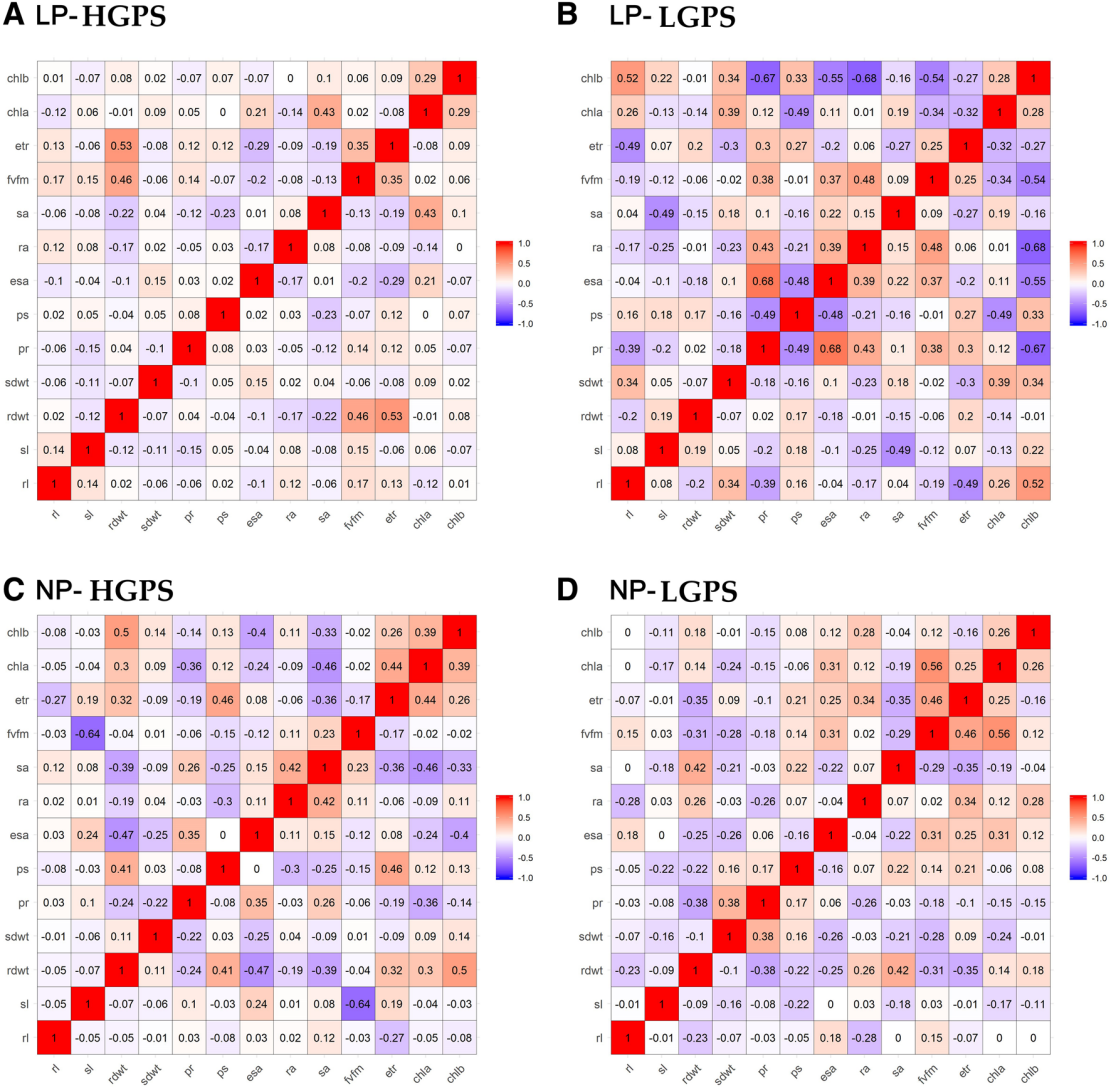
Correlogram of traits under LP and NP conditions in HGPS and LGPS at 24 days. The scale represents Pearson correlation values. Blue and red colours represent negative and positive correlation, respectively. root length (rl), shoot length (sl), root dry weight (rdwt), shoot dry weight (sdwt), P content in root (pr), P content in shoot (ps), P content in grain (pg), externally secreted acid phosphatase (esa), root acid phosphatase (ra), shoot acid phosphatase (sa), grain length (gl), grain width (gw), grain area (ga), (fvfm) electron transport rate (etr), chlorophyll A content (chla), chlorophyll B content (chlb).

At 24 days, LP-HGPS showed significant and positive correlation of germination%, root length, and shoot length with seedling vigour. FV/Fm and ETR showed significant positive correlation with root dry weight. Chl a showed significant positive correlation with SApase (Fig. 17A). In LP-LGPS, shoot length was negatively and significantly correlated with SApase, while Chl b was negatively correlated with Fv/Fm, RApase, ES Apase, and root P content. P content in root was positively and significantly correlated with ES Apase, RApase, and Fv/Fm. Shoot P content was negatively correlated with root P content, ES Apase, and Chl a (Fig. 17B). These correlations were not observed in NP-HGPS and NP-LGPS (Fig. 17C,D).

### Gene expression analysis

**Figure 18:**
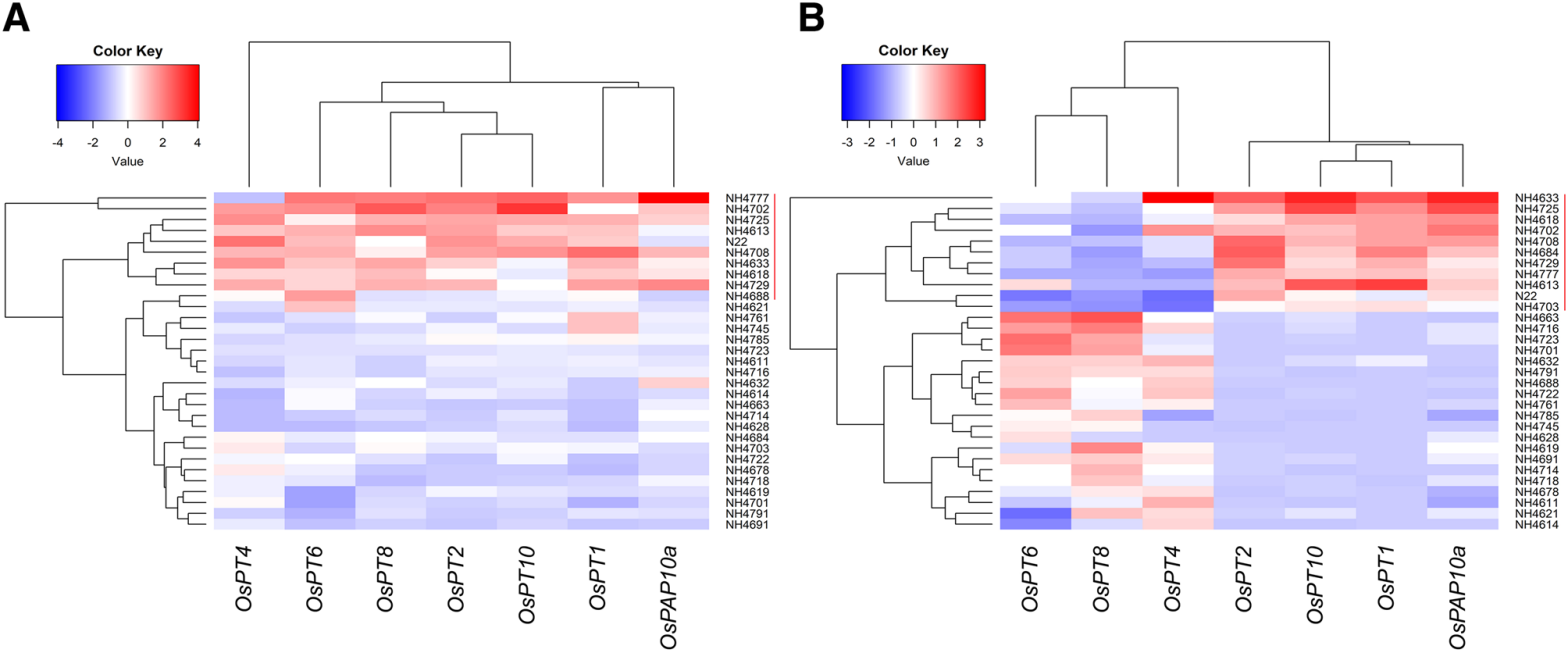
Heatmaps of relative expression levels of the genes involved in the P uptake and transport. Expression of these genes in seedlings obtained from germination of seeds harvested from low P plot was compared with the seedlings obtained from germination of seeds harvested from normal P plot. (**A**) Relative expression levels of genes in roots of 12 days old seedlings. (**B**) Relative expression levels of genes in roots of 24 days old seedlings. LGPS mutants and WT are shown by red bar.

**Figure 19:**
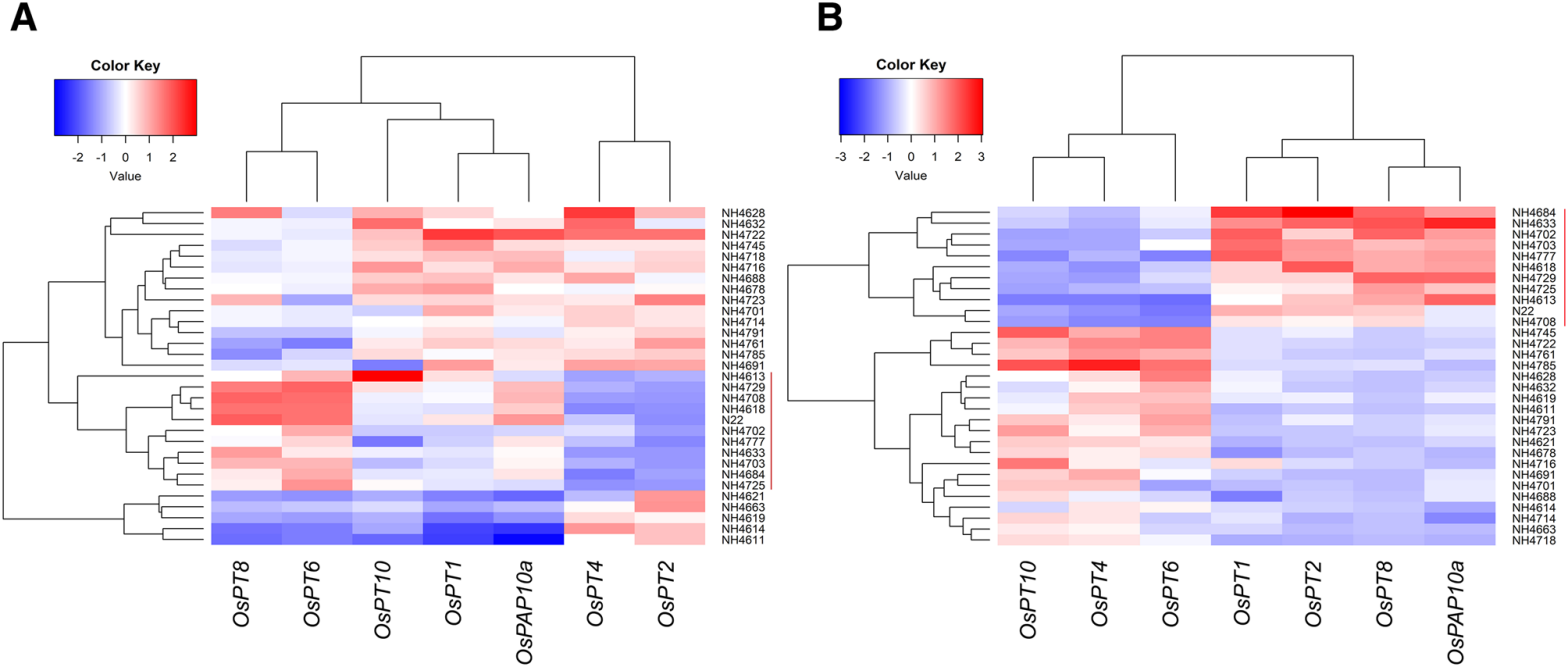
Heatmaps of relative expression levels of the genes involved in the P uptake and transport. Expression of these genes in seedlings obtained from germination of seeds harvested from low P plot was compared with the seedlings obtained from germination of seeds harvested from normal P plot. (**A**) Relative expression levels of genes in shoots of 12 days old seedlings. (**B**) Relative expression levels of genes in shoots of 24 days old seedlings. LGPS mutants and WT are shown by red bar.

To decipher the relative expression of phosphate starvation responsive (PSR) genes, a set of seven P transporter genes was selected based on their critical roles in P metabolism^28^. The expression level of all the genes was similar in NP-HGPS, NP-LGPS, and NP-N22. While comparing the expression level of genes in LP with NP, all the seven genes showed a distinct expression pattern among LGPS and HGPS. In root of 12 days old seedling, these genes showed up-regulation in all the LGPS, while in root of 24 days old seedlings, *OsPT6*, *OsPT8*, and *OsPT4* showed up-regulation in HGPS but *OsPT2,* and *OsPT10, OsPT1,* and *OsPAP10a* showed up-regulation in LGPS (Fig. 18). Similar analysis was performed in shoot tissue of 12- and 24-days old seedlings. At 12 days, shoot of HGPS showed up-regulation of *OsPT4, OsPT2* while LGPS showed up-regulation of *OsPT6*. Other genes did not show a distinct expression between LGPS and HGPS. At 24 days, shoot of HGPS showed up-regulation of *OsPT4* and *OsPT10* while LGPS showed up-regulation of *OsPT1, OsPT2, OsPT8,* and *OsPAP10a*. *OsPT6* did not show a distinct expression between LGPS and HGPS (Fig. 19).

## Discussion

Phosphorus is an essential element of seed. While low P concentration in seeds can be detrimental to the seed germination, seedling establishment, and plant growth, the higher P concentration in seeds is undesirable due to several reasons: 1—Excess driving of soil P into seed P leading to P depletion from soil and increased use of P fertilizers, 2—Adverse effects of excess P to human body, especially the negative effect on uptake of other nutrients, 3—Increased P concentration in human waste pollute the environment. Therefore, understanding the minimum seed P concentration to maintain seedling vigour is crucial for sustainable use of P fertilizers in agriculture. It is necessary to design the breeding programs for development of cultivars loading optimum concentration of P in seeds. In this study, we utilized the EMS mutants of rice cultivar N22 to study the effect of seed P concentration on seedling vigour and associated physiological/biochemical processes. Further, we identified few mutants showing insignificant effect of reduced seed P concentration on seedling vigour.

Shoot/root length and dry weight of seedlings are affected adversely due to low P concentration in seeds. LP-HGPS showed better performance than LP-LGPS and wild type N22 at both the time points, however, the differences were more pronounced at 12 days than 24 days old seedlings, suggesting that external P supply may abridge the differences as the plant growth progresses. Earlier studies suggested that adverse effects of low P concentration of seeds on seedling vigour can be reduced by applying external soil P^12,15,16^. Germination percentage and SVI was more severely affected in LP-LGPS than LP-HGPS suggesting that initial seed P concentration does affect the early seedling vigour and germination even though P is supplemented in soil. A germination percentage of 85 and 74 was recorded in cases of LP-LGPS and LP-HGPS, respectively. Alpha-amylase enzyme activity was also more in LP-HGPS than LP-LGPS in 4 and 8 days old seedlings. Alpha-amylase activity directly influences seed germination by hydrolyzing the stored starch for nourishing the developing embryo^38^. Further, physiological parameters of LP-LGPS and LP-HGPS revealed that Chl a, Chl b, Fv/Fm, and ETR were also adversely affected in seedlings obtained from low P seeds. P deficiency disrupts the photosynthetic machinery and the electron transport chain^39^. P content in seeds has direct effect on seedling vigour and crop establishment as it affects root development and associated physiological processes^10–14^. 10–20% reduction of seedling biomass and P uptake was noticed in low P seed germinated in P-deficient soil^16^. It should be noted that these parameters were not different among NP-HGPS, NP-LGPS, and NP-N22 suggesting that effects are due to low P seed concentration only and not because of genetic mutation influencing physiological processes regulating these parameters.

The percent increase in acid phosphatases (ESApase, RApase, and SApase) from NP to LP conditions and enhanced activity of these enzymes in LGPS in comparison to HGPS indicate the phosphorus stress in early seedlings due to low P content in seeds. Increased activity of acid phosphatases has been reported under low P stress^40,41^. The activity of these enzymes was 10%, 47%, and 45% lower in 24 days old LP seedlings as compared to 12 days old seedlings suggesting that P stress experienced initially due to low P content in seeds gets subside as the growth progresses. Notably, Chl a was decreased in 12 days but increased in 24 days old seedlings obtained from low P seeds, suggesting plants attempt to compensate the initial losses due to low P. Phosphorus deficiency has a negative impact on the photosynthetic characteristics of rice^42^. Similar observation in case of root and shoot P content was observed. Initially at 12 days, LP-HGPS showed 13% and 27% more root and shoot P content than LP-LGPS but this gap was reduced to 10% and 14% at 24 days. Seed P reserves are critical for root development of seedling that influences acquisition of soil nutrients^11,14^. The early vigour trait contributes to improved P uptake in plants^43^. Therefore, seed P concentration affects seedling vigour and later seedling vigour influences P uptake by plants.

The 24 days old seedlings of mutant *NH4614* showed similar shoot length while *NH4785* showed similar SVI from harvested seeds of low P and normal P plots. The grain/seed P content of *NH4614* and *NH4785* was 1.88 mg/g DW and 1.92 mg/g DW in low P plot harvested seeds and, 2.80 mg/g DW and 2.90 mg/g DW in normal P plot harvested seeds, respectively. Among all the mutants, root P content of LP-*NH4785* at 24 days was least (0.513 mg/g DW) and shoot P content was one among the highest (2.35 mg/g DW), suggesting that this mutant may have efficient P transport system active at early growth stage. Notably, LP-*NH4791* showed maximum root dry weight (0.027 g/plant) while LP-*NH4785* and LP-*NH4714* showed root dry weight of 0.026 g/plant and 0.025 g/plant at 24 days, suggesting that ability to develop robust roots in early growth stages make these mutants less vulnerable to low grain P. *NH4791* showed similar SVI at 24 days and less affected SVI at 12 days in seeds obtained from low P when compared with seeds obtained from normal P field plots. Not only SVI, even Chl b content of 12 days old seedlings of *NH4785* and *NH4791* was similar in seeds obtained from low P and normal P field plots. The highest Fv/Fm (0.71) was recorded in 24 days old seedling of LP-*NH4791*. LP-*NH4785* and LP-*NH4714* showed Fv/Fm as 0.70 and 0.69 at 24 days. *NH4714* at 24 days showed 2nd highest SVI (after *NH4791*) in seeds obtained from low P field. The grain P content of *NH4791* and *NH4714* was 1.84 mg/g DW and 1.94 mg/g DW in low P plot harvested seeds and, 2.84 mg/g DW and 2.92 mg/g DW in normal P plot harvested seeds, respectively. LP-*NH4663* (grain P content 1.64 mg/g DW) showed fastest recovery of Chl a, while *NH4618* showed similar level of Chl a in 24 days old seedlings obtained from seeds of low P (grain P content 1.10 mg/g DW) and normal P (grain P content 2.86 mg/g DW) field plots. Detailed data of such selected mutants is summarized in Tables S1 and S2. These identified low seed-P mutants having ability to compensate for lower endogenous supply of P from the seed are having desirable attributes to minimize the loss of soil P in the form of harvested plant product. Associated genetic factors and linked genetic markers of these mutants can be investigated, and these can be used to produce breeding lines that can exhibit rapid root development and seedling vigour even with reduced seed P concentration. These efforts are needed to achieve the goal of reducing the twofold negative impact of unsustainable high P fertilizer applications, and conserving the reserves of rock-phosphate resources for sustainable agriculture^9^. Decreasing the 20% grain P concentration in rice can reduce depletion of 0.4 Mt P worth of $2 billion P fertilizers every year globally^44^. The mutants like *NH4785* and *NH4791* showed ~ 35% reduction in grain P content of low P plot harvested seeds than normal P plot harvested seeds, however, the seedling vigour and other physiological processes of next generation seedlings were unaffected due to low grain P.

While comparing the P transporter genes expression in roots, all the 7 transporter genes showed up-regulation in 12 days old LGPS of low P than LGPS of normal P, suggesting that low P content in seeds triggered the expression of genes involved in P transport at a very early stage. At 24 days, *OsPT2,* and *OsPT10, OsPT1,* and *OsPAP10a* showed up-regulation in LGPS while *OsPT6*, *OsPT8*, and *OsPT4* showed up-regulation in HGPS suggesting that P transporters are differentially regulated in seeds with different P content. Similar observations were made in shoot tissue. At 12 days, LGPS showed up-regulation of *OsPT6* but HGPS showed up-regulation of *OsPT4, OsPT2*. At 24 days, *OsPT4* and *OsPT10* were upregulated in HGPS while *OsPT1, OsPT2, OsPT8,* and *OsPAP10a* were up-regulated in LGPS. *OsPT1* and *OsPT8* are involved in P uptake from roots^45,46^ while *OsPT2* is involved in transportation of P from root to shoot^47^. Both of these transporters were active at 2 days after germination suggesting that the transport machinery of P became active at a very early stage of seedling development^9^. While some of the P transporters such as *OsPT2, OsPT4* and *OsPT8* are well characterized^44,47–51^, function of several other transporter genes in P homeostasis needs to be deciphered. Initial seed P concentration has significant influence on regulation of P transporters that appears to be highly dynamic.

In 12 day old seedlings, a positive correlation between root dry weight and grain P content was observed in LP-HGPS. In case of LP-LGPS, a positive correlation between root length and RApase, root dry weight and Chl b content, P content in root and Chl b content was observed. In 24 dasy old seedlings, a positive correlation between germination percent, root length, shoot length and seedling vigour was observed in LP-HGPS. Further, root dry weight showed positive correlation with Fv/Fm and ETR. In LP-LGPS, root P content was positively and significantly correlated with ES Apase, RApase, and Fv/Fm. Such correlations observed under low P further indicate that seed P content influences physiological and metabolic processes of seedlings which are interconnected. These correlations did not exist in NP-HGPS and NP-LGPS.

This study demonstrated that P concentration in seeds have significant influence on seedling vigour and associated physiological processes. It has immense importance in addressing the earlier non-conclusive reports on effects of seed P concentration on seedling vigour. We suggest that these mutants are appropriate genetic material to resolve the longstanding scientific question. The mutants such as *NH4614*, *NH4785*, *NH4791*, *NH4714*, *NH4663*, and *NH4618* are appropriate genetic resources to develop breeding lines for low P grains which can help achieving the goal of sustainable P use in agriculture. Many of the mutants identified in this study can be utilized for understanding the molecular and biochemical basis of seed P role in germination, early seedling vigour, and crop establishment. We believe that these mutant lines can be used to produce the hybrids/inbreds that can help to reduce the loss of soil P in the long run. Pariasca-Tanaka et al.^17^ suggested that negative effect of low concentration of P in seed on seedling vigour could be genotype-specific and it is possible to mitigate the negative effect by selection and breeding of appropriate rice lines loading lesser P in seeds yet exhibiting good seedling vigour and growth. The outcome of present study highlights the value of mutations and causal genes that can reduce the dependency on finite P resources.

## Supplementary Information

Supplementary Information.
