# Supplementary material for: Revealing the effect of seed phosphorus concentration on seedling vigour and growth of rice using mutagenesis approach
Source: Sci Rep. 2022 Jan 24;12:1203. doi: 10.1038/s41598-022-04983-9 (PMC8786825; doi:10.1038/s41598-022-04983-9)
Supplement: Supplementary file 1 — Supplementary Information. [file 41598_2022_4983_MOESM1_ESM.docx]

**Revealing the effect of seed phosphorus concentration on seedling vigour and growth of rice using mutagenesis approach**

P. Yugandhar^1a^, N. Veronica^1a^, D. Subrahmanyam^1^, P. Brajendra^1^, S. Nagalakshmi^1^, Akanksha Srivastava^1^, S.R. Voleti^1^, N. Sarla^1^, R.M. Sundaram^1^, Amitha Mithra Sevanthi V^2^, A.K. Singh^3^ and Satendra K Mangrauthia^1^*

*^1^ICAR-Indian Institute of Rice Research, Hyderabad, India*

*^2^ICAR-National Institute for Plant Biotechnology, New Delhi, India*

*^3^ICAR-Indian Agricultural Research Institute, New Delhi, India*

^a^ contributed equally.

*To whom correspondence should be addressed.

Satendra K. Mangrauthia

ICAR-Indian Institute of Rice Research, Hyderabad-500030, India

Email: Satendra.KM@icar.gov.in

Phone: +91-40-24591342

Orcid ID: 0000-0001-6316-9848


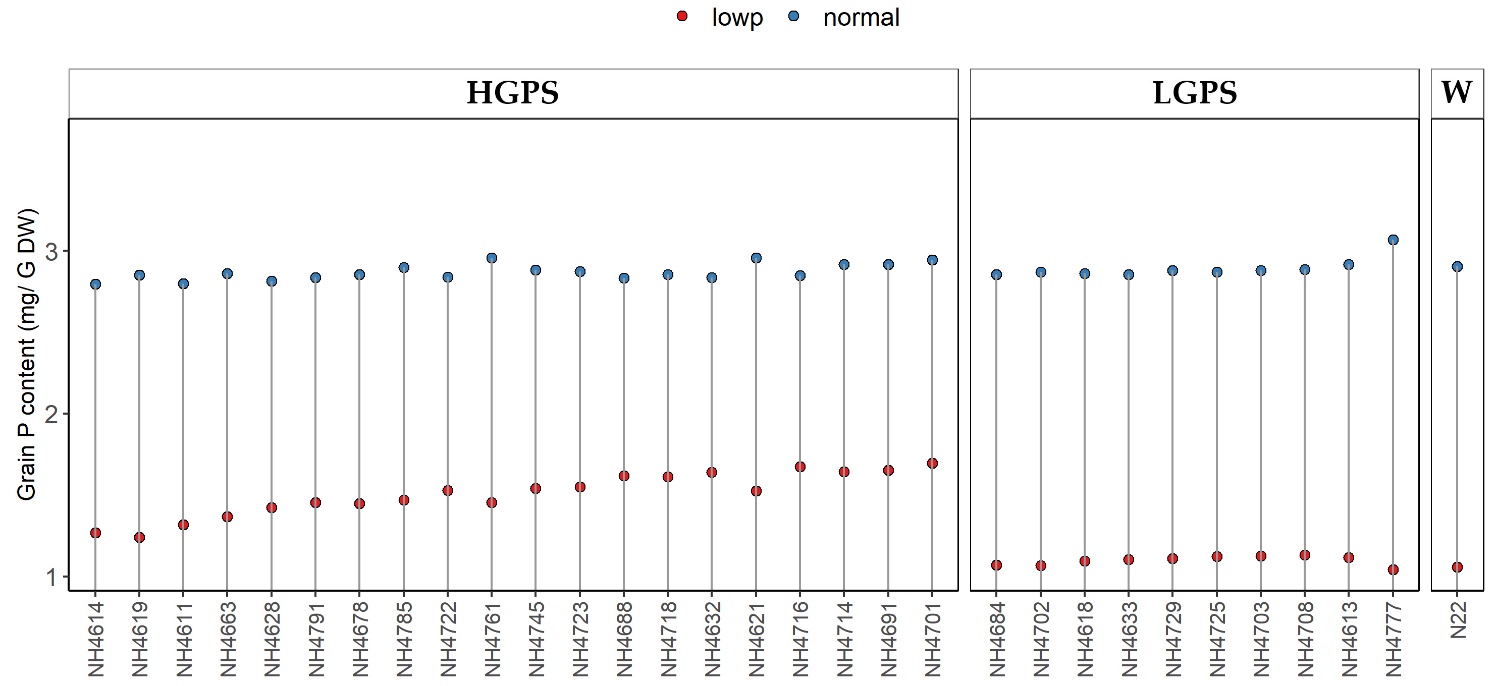


**Fig. S1.** Initial grain P concentration in seeds collected from low P (shown in red dots) and normal P (shown in blue dots) field plots.

Table S1. Comparative analysis of different seedling, physiological and biochemical traits in best mutants at 12 days in low P and normal conditions.

|  | Root length | | Shoot length | | Root dry weight | | Shoot dry weight | | Seedling vigour | | Chlorophyll A content | | Chlorophyll B content | | Root P content | | Shoot P content | | Grain P content | | ES Apase | | Root Apase | | Shoot Apase | | Alpha-amylase activity | |
| --- | --- | --- | --- | --- | --- | --- | --- | --- | --- | --- | --- | --- | --- | --- | --- | --- | --- | --- | --- | --- | --- | --- | --- | --- | --- | --- | --- | --- |
| Mutant | P+ | P- | P+ | P- | P+ | P- | P+ | P- | P+ | P- | P+ | P- | P+ | P- | P+ | P- | P+ | P- | P+ | P- | P+ | P- | P+ | P- | P+ | P- | P+ | P- |
| NH4791 | 8.60 | 11.00 | 11.40 | 9.40 | 0.02 | 0.02 | 0.05 | 0.03 | 1872.20 | 1876.80 | 1.32 | 1.23 | 0.55 | 0.53 | 3.52 | 2.35 | 3.86 | 3.19 | 2.84 | 1.46 | 1.36 | 3.13 | 1.36 | 4.52 | 1.74 | 3.83 | 10.61 | 7.30 |
| NH4785 | 9.60 | 11.40 | 12.20 | 7.60 | 0.02 | 0.02 | 0.05 | 0.03 | 2027.00 | 1755.40 | 1.34 | 1.11 | 0.55 | 0.53 | 3.37 | 2.40 | 3.87 | 3.42 | 2.90 | 1.47 | 1.46 | 3.20 | 1.41 | 4.40 | 1.75 | 3.84 | 10.67 | 7.45 |
| NH4714 | 9.80 | 11.60 | 12.00 | 8.20 | 0.02 | 0.02 | 0.05 | 0.03 | 2062.60 | 1825.60 | 1.36 | 1.16 | 0.63 | 0.50 | 3.25 | 2.44 | 3.89 | 3.28 | 2.92 | 1.64 | 1.29 | 3.15 | 1.56 | 4.36 | 1.78 | 3.93 | 10.57 | 7.37 |
| NH4663 | 8.00 | 12.60 | 12.60 | 8.20 | 0.02 | 0.02 | 0.05 | 0.03 | 1969.80 | 1684.60 | 1.36 | 1.15 | 0.53 | 0.47 | 3.40 | 2.24 | 3.91 | 3.24 | 2.86 | 1.37 | 1.44 | 3.20 | 1.53 | 4.48 | 1.79 | 3.87 | 10.84 | 7.45 |
| NH4614 | 7.60 | 10.80 | 12.20 | 6.20 | 0.02 | 0.01 | 0.05 | 0.03 | 1881.00 | 1272.00 | 1.37 | 1.04 | 0.65 | 0.43 | 3.32 | 2.10 | 3.77 | 2.50 | 2.86 | 1.10 | 1.34 | 3.22 | 1.54 | 5.28 | 1.68 | 4.48 | 10.66 | 5.21 |
| NH4618 | 8.40 | 10.60 | 12.40 | 9.40 | 0.02 | 0.01 | 0.05 | 0.03 | 1980.40 | 1663.60 | 1.33 | 1.19 | 0.57 | 0.44 | 2.96 | 2.17 | 3.92 | 3.51 | 2.80 | 1.27 | 1.27 | 3.25 | 1.54 | 4.58 | 1.84 | 3.79 | 10.70 | 7.57 |
| N22 | 8.40 | 13.60 | 12.60 | 8.00 | 0.02 | 0.01 | 0.05 | 0.03 | 1953.40 | 1555.00 | 1.38 | 0.99 | 0.56 | 0.39 | 3.42 | 2.06 | 3.61 | 2.36 | 2.85 | 1.06 | 1.26 | 3.17 | 1.36 | 5.77 | 1.75 | 4.31 | 10.46 | 5.12 |

**Table S2.** Comparative analysis of different seedling, physiological and biochemical traits in best mutants at 24 days in low P and normal conditions.

|  | Root length | | Shoot length | | Root dry weight | | Shoot dry weight | | Seedling vigour | | Fv/Fm | | ETR | | Chlorophyll A content | | Chlorophyll B content | | Root P content | | Shoot P content | | ES Apase | | Root Apase | | Shoot Apase | | Alpha-amylase activity | |
| --- | --- | --- | --- | --- | --- | --- | --- | --- | --- | --- | --- | --- | --- | --- | --- | --- | --- | --- | --- | --- | --- | --- | --- | --- | --- | --- | --- | --- | --- | --- |
| Mutant | P+ | P- | P+ | P- | P+ | P- | P+ | P- | P+ | P- | P+ | P- | P+ | P- | P+ | P- | P+ | P- | P+ | P- | P+ | P- | P+ | P- | P+ | P- | P+ | P- | P+ | P- |
| NH4791 | 12.60 | 16.60 | 25.60 | 22.00 | 0.03 | 0.03 | 0.07 | 0.05 | 3575.40 | 3550.40 | 0.78 | 0.71 | 27.62 | 22.50 | 1.74 | 1.79 | 0.83 | 0.61 | 2.56 | 1.77 | 3.15 | 2.25 | 1.34 | 2.81 | 1.22 | 2.49 | 1.42 | 2.21 | 6.14 | 5.59 |
| NH4785 | 11.60 | 15.60 | 25.00 | 21.80 | 0.03 | 0.03 | 0.07 | 0.05 | 3396.20 | 3455.40 | 0.77 | 0.70 | 27.40 | 22.00 | 1.66 | 1.85 | 0.82 | 0.56 | 2.49 | 1.13 | 3.15 | 2.35 | 1.29 | 2.90 | 1.19 | 2.45 | 1.40 | 2.15 | 6.13 | 5.62 |
| NH4714 | 12.00 | 17.00 | 25.20 | 23.60 | 0.03 | 0.03 | 0.07 | 0.05 | 3519.80 | 3743.20 | 0.76 | 0.69 | 26.36 | 21.16 | 1.72 | 1.76 | 0.75 | 0.57 | 2.60 | 1.85 | 3.12 | 2.36 | 1.41 | 2.87 | 1.11 | 2.67 | 1.37 | 2.19 | 6.20 | 5.54 |
| NH4663 | 11.60 | 16.60 | 25.20 | 23.40 | 0.03 | 0.02 | 0.07 | 0.05 | 3518.00 | 3239.60 | 0.76 | 0.69 | 26.38 | 21.84 | 1.57 | 1.86 | 0.75 | 0.59 | 2.73 | 1.84 | 3.05 | 2.23 | 1.44 | 2.85 | 1.21 | 2.56 | 1.51 | 2.33 | 6.16 | 4.62 |
| NH4614 | 11.60 | 14.60 | 25.20 | 23.00 | 0.03 | 0.02 | 0.07 | 0.05 | 3495.40 | 2811.00 | 0.78 | 0.65 | 26.26 | 17.40 | 1.75 | 1.74 | 0.76 | 0.55 | 2.43 | 1.59 | 3.21 | 2.03 | 1.15 | 2.85 | 1.20 | 2.53 | 1.25 | 2.25 | 6.21 | 4.76 |
| NH4618 | 12.20 | 15.20 | 24.00 | 23.60 | 0.03 | 0.02 | 0.07 | 0.05 | 3447.20 | 3227.80 | 0.76 | 0.70 | 25.70 | 20.68 | 1.62 | 1.78 | 0.76 | 0.54 | 2.51 | 1.64 | 3.04 | 2.24 | 1.24 | 2.85 | 1.24 | 2.67 | 1.56 | 2.29 | 6.16 | 5.46 |
| N22 | 11.20 | 15.80 | 25.60 | 23.80 | 0.03 | 0.02 | 0.06 | 0.05 | 3421.80 | 2851.80 | 0.78 | 0.63 | 25.74 | 16.38 | 1.77 | 1.83 | 0.77 | 0.73 | 2.31 | 1.32 | 3.16 | 2.06 | 1.20 | 2.78 | 1.25 | 2.27 | 1.26 | 2.17 | 6.17 | 4.59 |
